# Glutamatergic Neurons in the Amygdala Are Involved in Paclitaxel-Induced Pain and Anxiety

**DOI:** 10.3389/fpsyt.2022.869544

**Published:** 2022-04-14

**Authors:** Jiaxin Liu, Dangchao Li, Jing Huang, Jing Cao, Guohong Cai, Yuexian Guo, Guiying Wang, Shuang Zhao, Xiuli Wang, Shengxi Wu

**Affiliations:** ^1^Department of Anesthesiology, Third Affiliated Hospital of Hebei Medical University, Shijiazhuang, China; ^2^Department of Neurobiology, School of Basic Medicine, Fourth Military Medical University, Xi'an, China; ^3^Department of Surgery, Third Affiliated Hospital of Hebei Medical University, Shijiazhuang, China

**Keywords:** paclitaxel, chemotherapy pain, amygdala, anxiety, glutamatergic neuron

## Abstract

Paclitaxel is widely used as a first-line chemotherapy agent to treat malignant tumors. However, paclitaxel causes peripheral nerve fiber damage and neuropathic pain in some patients. In addition, patients received paclitaxel chemotherapy are often accompanied by negative emotions such as anxiety. The amygdala is critically involved in regulating pain signals, as well as anxiety. The purpose of this study is to clarify the role of Ca^2+^/calmodulin-dependent protein kinase II (CaMKII)-positive glutamatergic neurons in the amygdala in paclitaxel-induced pain and negative affective symptoms. Intraperitoneal injection of paclitaxel into mice caused mechanical and thermal allodynia, as measured by Von Frey test and Hargreaves test, and anxiety, as measured by open field test and elevated plus maze test. Immunofluorescence staining revealed that c-fos-positive neurons were significantly more in the basolateral amygdala (BLA) and central amygdala (CeA) in paclitaxel-treated mice than untreated mice. Furthermore, part of c-fos-positive neurons in the BLA were immunoreactive of CaMKII. Engineered Designer receptors exclusively activated by designer drugs (DREADD) receptor hM4Di or hM3Dq was selectively expressed on CaMKII neurons by injection of adeno-associated virus (AAV) vectors containing CaMKII and hM4Di or hM3Dq. Administration of DREADD agonist CNO to selectively inhibit the CaMKII neurons in the BLA significantly increased the paw withdrawal thresholds and paw withdrawal latencies. In addition, selectively inhibition of CaMKII neurons in the BLA alleviated anxiety behavior without affecting the motor activity. In summary, our findings suggest that CaMKII neurons in the amygdala are critical for neuropathic pain and anxiety behaviors induced by paclitaxel chemotherapy.

## Introduction

Paclitaxel is a tetracyclic diterpene compound that is widely used to treat variety of carcinomas such as ovarian and breast cancers, lung cancer, colorectal cancer, melanoma, head and neck cancer, lymphoma, and brain tumors (1, 2). Paclitaxel causes mitochondrial damage and cell apoptosis via inhibiting the dynamic instability of microtubules (3). Paclitaxel exerts its antitumor effects accompanied by various side effects such as chronic pain with negative emotions, impairment of cognitive function (chemo brain), and neuroinflammation (4, 5). Paclitaxel-induced neuropathy manifests as mechanical and sensory allodynia, numbness, and tingling (6–8), which may easily induce anxiety and depression-like emotions (9, 10). It has been shown that intraperitoneal injection of paclitaxel accumulatively produced anxiety and depression-like emotions without affecting the weight and exercise capacity of the mice (9). However, the precise mechanisms underlying paclitaxel-induced chronic pain with negative emotions are still need to be elucidated.

Although paclitaxel-induced peripheral neuropathic pain can be treated using analgesics and antidepressants, these medicines are often inadequately effective and induces many side effects (11, 12) including alteration of neurocircuitry. For instance, paclitaxel treatment alter function of brain areas involved in the emotional and motivational response to chronic pain (13). Furthermore, paclitaxel treatment induces negative emotions and reduces NR1 levels in the prefrontal cortex (PFC) (14). Since the PFC receives glutamatergic projections from the basolateral amygdala (15), we speculate that the amygdala is also involved in the regulation of paclitaxel chemotherapy pain.

As a part of the limbic system, the amygdala is an almond-shaped structure located in the medial temporal lobe and plays a critical role in feeling, learning, fears, anxiety, and depression (16–19). The amygdala consists of anatomically and functionally different nuclei (20–23) including basolateral amygdala (BLA), central nucleus (CeA) and intervening cell clusters between BLA and CeA (24). BLA has cortical characteristics, contains Ca^2+^/calmodulin-dependent protein kinase II (CaMKII) excitatory output neurons (25–27). BLA integrates sensory information and sensory-related emotions from the cortex and conveys them to the CeA (17, 24, 28). BLA neurons project to medial PFC, ACC (Anterior cingulate cortex), peripheral cortex, and insular cortex (29–34). It has been shown that the BLA is involved in the regulation of pain signal (24, 35–37) and the BLA has a large number of CaMKII-positive glutamatergic neurons projecting to mPFC, which plays an important role in the pain-induced mPFC inactivation (32, 33, 35, 38–40). The CeA integrates nociceptive information with multi-modal information about the internal and external environment of the body, and is the main output nucleus of the amygdala function (21, 24). Through associative processing, the LA/BLA attaches emotional-affective content to the sensory inputs and transmits that highly processed information to the amygdala output region in the CeA for further processing as part of amygdala fear and anxiety circuitry. This LA/BLA-CeA projection is now known to generate and modulate pain-related negative emotional behaviors (24). Recent studies have confirmed that the functional conversion of DOR1 and DOR2 is related to the state of anxiety in different pain stages by regulating the activity of specific pathways (BLA-CeA and PBN-CeA) (41). However, the role of glutamatergic neurons in amygdala in paclitaxel-induced pain and anxiety is unknown. Therefore, in the present study, by using paclitaxel chemotherapy pain model and chemogenetic method, we explored the role of CaMKII-positive glutamatergic neurons in paclitaxel-induced pain and anxiety.

## Materials and Methods

### Animals

C57BL/6 mice aged 8 to 12 weeks were obtained from the animal facility of the Fourth Military Medical University and maintained at an ambient temperature of 25°C, with a 12 h light and 12 h dark cycle. The mice were given *ad libitum* access to food and water. All animal operations were approved by the Experimental Animal Management Ethics Committee of the Fourth Military Medical University.

### Establishment of Paclitaxel Chemotherapy Pain Model

Paclitaxel (PTX, TCI, P1632) was dissolved in Cremopher EL (CEL, SIGMA, 61791-12-6) and absolute ethanol diluted in equal proportions to a concentration of 6mg/ml and then frozen at −20°C. The working solution was diluted with normal saline to 0.4 mg/ml; the vehicle group was given the Cremopher EL and absolute ethanol in equal proportions and diluted with the normal saline to an equal volume. Paclitaxel (4 mg/kg, intraperitoneal injection) was given daily for 8 days (9). The volume is 6.67 ml/kg (cumulative dose: 32 mg/kg, intraperitoneal injection) to induce neuropathy.

### Von Frey Test

Three days before the experiment, the mice were placed on a metal iron frame with a transparent box at the same time period as the experiment to adapt to the experimental environment for one hour. On the day of the experiment, the mice need to adapt to the metal grid for one hour. After the mouse stopped grooming and other behaviors, Von-Frey fiber filaments weighing 0.008 to 2.0 g were used to stimulate the palms of the rear plantar of the mice, and the fiber filaments were bent in an S shape for 3 to 5 s. A positive response was defined as an obvious rapid foot lifting or licking due to pain. Movement or foot lifting due to other discomfort is considered negative. There should be an interval of at least 20 s between each plantar stimulation or wait for the mouse returns to a calm state. After five tests, three positive weights can be regarded as the paw withdrawal thresholds of the mouse.

### Hargreaves Test

The mice were placed on a 30°C constant temperature plate with a transparent box and adapted to the experimental environment for one hour every day at the same time period as the experiment for three days before the experiment day. Before the start of the experiment, set the required heat pain intensity and the longest heat pain stimulation time. On the day of the experiment, it is necessary for the mice to adapt to the metal grid for one hour. After the mouse stopped grooming and other behaviors, a thermal stimulator was used to illuminate the palm area of the rear plantar of the mouse. The longest irradiation time should not exceed 20 s to avoid burns on the soles of the mice or causing hyperalgesia. The sign to stop the timer was defined as an obvious rapid foot-lifting or licking response. A total of 10 times of mouse foot lifting time were recorded, and the average value of 10 times was the paw withdrawal latencies of this mouse.

### Open Field Test

The mice were put into the laboratory one hour before the experiment to adapt to the experimental environment. Wipe the open field area with a length of 40 cm each with a 50% ethanol solution to prevent the mice from being affected by other odors. Afterwards, the mice were gently placed in the central area of the open field, and then a 5 min travel trajectory of each mouse was recorded by video.

### Elevated Plus Maze Test

The mice were put into the laboratory one hour before the experiment to adapt to the experimental environment. Wipe the elevated plus maze with a 50% ethanol solution to prevent the mice from being affected by other odors. Then gently place the mouse at the center of the junction area between the open arm and the closed arm, and then start to record the trajectory of the mouse by video for five minutes for each mouse.

### Stereotactic Injection in the Brain

The mice were anesthetized by intraperitoneal injection of 5% chloral hydrate. After the mice were anesthetized, the scalp was prepared and the incisors were fixed to the rack of the operating table, and the ear rack was used to fix the head of the mouse in the external auditory canal. The fontanel was used as a reference to level the head. The BLA is M/L = ±3.00, A/P = −1.25, D/V = −4.75. 250 nl of rAAV-CaMKIIa-hM4D(Gi)-EGFP-WPRE-pA (Cat#: PT-0524, titer: 5.54 × 10^12^ vg/mL, BrainVTA), rAAV-CaMKII-hM3D(Gq)-mCherry-WPREs (Cat#: PT-0049, titer: 5.33 × 10^12^ vg/mL, BrainVTA) and rAAV-CaMKIIa-EYFP-WPRE-pA (Cat#: PT-0107, titer: 2.50 × 10^12^ vg/mL, BrainVTA) were injected to each of the bilateral BLA of the three groups respectively for 5 min and the needle need to be left for 10 min. Then withdraw the needle, surgically sutured the skin of the skull, and the other experimental operations need to be wait for three weeks after the virus infected the neurons.

### Perfusion and Extraction

The mice were anesthetized by intraperitoneal injection of 5% chloral hydrate. After the mice were anesthetized, they were fixed on the surgical board in the supine position, the chest cavity was opened, and the heart was freed. Pierce the rounded needle into the left ventricle, and then pierce the right atrial appendage with scissors. 0.9% saline was quickly injected into the left ventricle by a 20 ml syringe. Then fixed with the titration 50 ml of 4% paraformaldehyde. Next, carefully peel off the skull with tweezers and take out the entire brain. The taken-out brain was immersed in 4% paraformaldehyde for post-fixation for four hours, and then the brain was placed in a 30% sucrose solution for dehydration at 4°C overnight. When the floating brain sinks into the solution, dehydration is complete.

### Immunofluorescence Histochemical Staining

Take out the dehydrated brain, cut it perpendicularly to the missing seam, place it on the stage, and embed it in OCT. Using a cryostat (Leica CM1950), put it in a microtome and quickly freeze it at −20°C for 20 min. Then transfer it to the slicing table, perform coronal sectioning with a thickness of 30 um, gently pick it out with a brush, and place it in a six-well plate of 0.1 M PBS. Three sets of brain slices were selected for each group, and rinsed for three times at room temperature with 0.1MPBS (pH 7.4) for 10 min each time. Then place the brain slices in the antibody diluent (3% BSA, 0.3% Triton X-100 PBS) that has been added to the primary antibody, and incubate at room temperature for 16 h on a shaker. Use the following primary antibodies: mouse anti c-fos (1:500; abcam, ab11959), rabbit anti CaMKII (1:200; abcam, ab5683). The brain slices were rinsed three times with 0.1MPBS at room temperature for 10 min each time, and then the brain slices were placed in the antibody diluent with the added secondary antibody, incubated for 4 h, and dapi was added at 3.5 h. Use the following secondary antibodies: Donkey Anti-Rabbit IgG H&L Alexa Fluor® 488 (1:500; invitrogen, A-21206), Goat Anti-Mouse IgG H&L Alexa Fluor® 594 (1:500; invitrogen, A-21202), DAPI (1:1000; Sigma, D9542). Then the brain slices were rinsed for three times with 0.1MPBS for 10 min each time.

### Pharmacogenetic Manipulation

Dissolve Clozapine N-oxide (CNO, TOCRIS) with 0.9% saline to a concentration of 0.25 mg/ml. One mg/kg of it was injected intraperitoneally to the three groups of mice (The hM4D, hM3D, and EYFP) 1 h before the behavioral test. CaMKII neurons in virus-transfected mice were activated or inhibited by CNO pharmacology.

### Image Acquisition and Statistical Analysis

Digital images were captured by FLUOVIEW software (FV10-ASW 1.7, Olympus) and saved in tiff image files. The data were presented as the mean ± *S.E.M*. For behavioral test data, repeated measures ANOVA was performed to detect overall differences followed by Bonferroni *post hoc* analysis for multiple comparisons. Student's *t*-test was used to determine the differences between groups. We performed a Shapiro-Wilk test prior to using any parametric statistical test, to ensure that the data follow a normal distribution. For data that do not follow a normal distribution, we used Mann Whitney test and Rank Sum test. *P* < 0.05 was regarded as statistically significant. GraphPad Prism 8 Software (GraphPad, USA) was used for all statistical analyses and statistical graph drawing.

## Results

### Establishment of Paclitaxel Chemotherapy Pain Model

**Figure 1:**
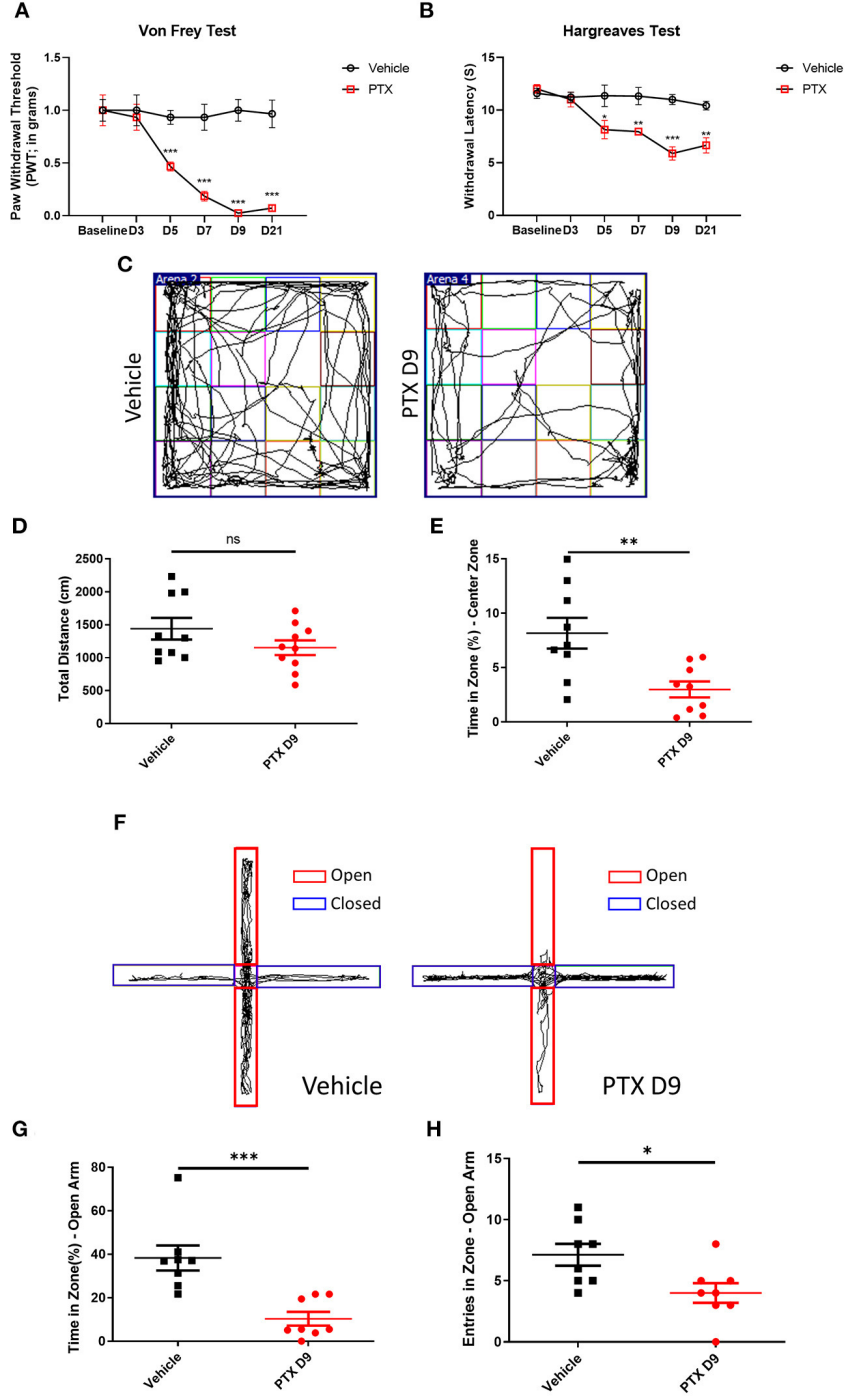
Establishment of paclitaxel chemotherapy pain model. **(A,B)** The behavioral changes of paw withdrawal thresholds and paw withdrawal latencies in the vehicle group and paclitaxel group at different time. **(C)** Open field test trajectory diagram of vehicle and paclitaxel groups. **(D)** Total distance statistics of the vehicle group and paclitaxel group. **(E)** The statistical results of the percentage of central area activity time in the vehicle group and paclitaxel group. **(F)** The trajectory diagram of the elevated plus maze test in the vehicle and paclitaxel groups. **(G)** Statistic results of the percentage of open arm entry time in the vehicle group and paclitaxel group. **(H)** Statistic results of the number of entries open arm in the vehicle group and paclitaxel group. *n* = 6–9, ns, no significant, *P < 0.05, **P < 0.01, ***P < 0.001.

To establish a paclitaxel chemotherapy pain model, paclitaxel dissolved in Cremopher EL and normal saline was injected intraperitoneally at 4 mg/kg body weight for 8 days. Paclitaxel administration significantly reduced the withdrawal threshold in Von Frey testing and thermal threshold in Hargreaves Test 3 to 21 days post injection (Figures 1A,B). The paclitaxel-treated mice and vehicle-treated mice were subjected to an open field and an elevated plus maze (EPM) test on day 9. The paclitaxel-treated mice spent significantly less time in the central area than the vehicle group (Figures 1C–E). In the EPM testing, the number of open-arm entries and the percentage of total open-arm staying time in EPM test were significantly less than those in vehicle group (Figures 1F–H).

**Figure 2:**
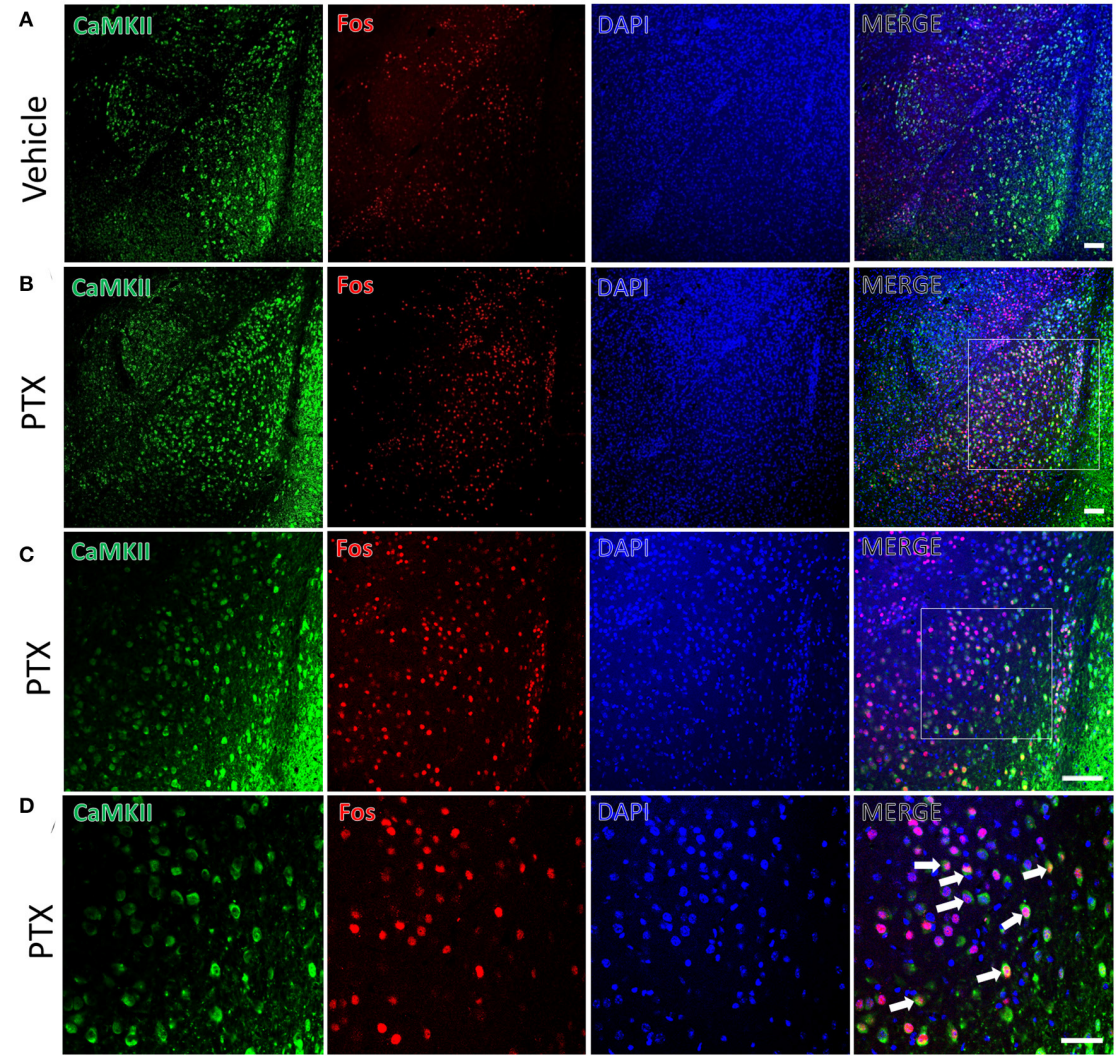
CaMKII neurons in the BLA are activated in paclitaxel-treated mice. **(A)** The expression of CaMKII (green) and Fos (red) in the BLA of the vehicle group. **(B,C)** The expression of CaMKII (green) and Fos (red) in the BLA of the paclitaxel group under different multiples microscope. **(D)** The magnified images of the rectangles indicated in **(C)**. Arrows indicate part of CaMKII and Fos double-labeled neurons. n=3 mice per group, 2 sections per mouse. Scale bars = 100 μm in **(A)** [applies to **(B,C)**]; 50 μm in **(D)**.

**Figure 3:**
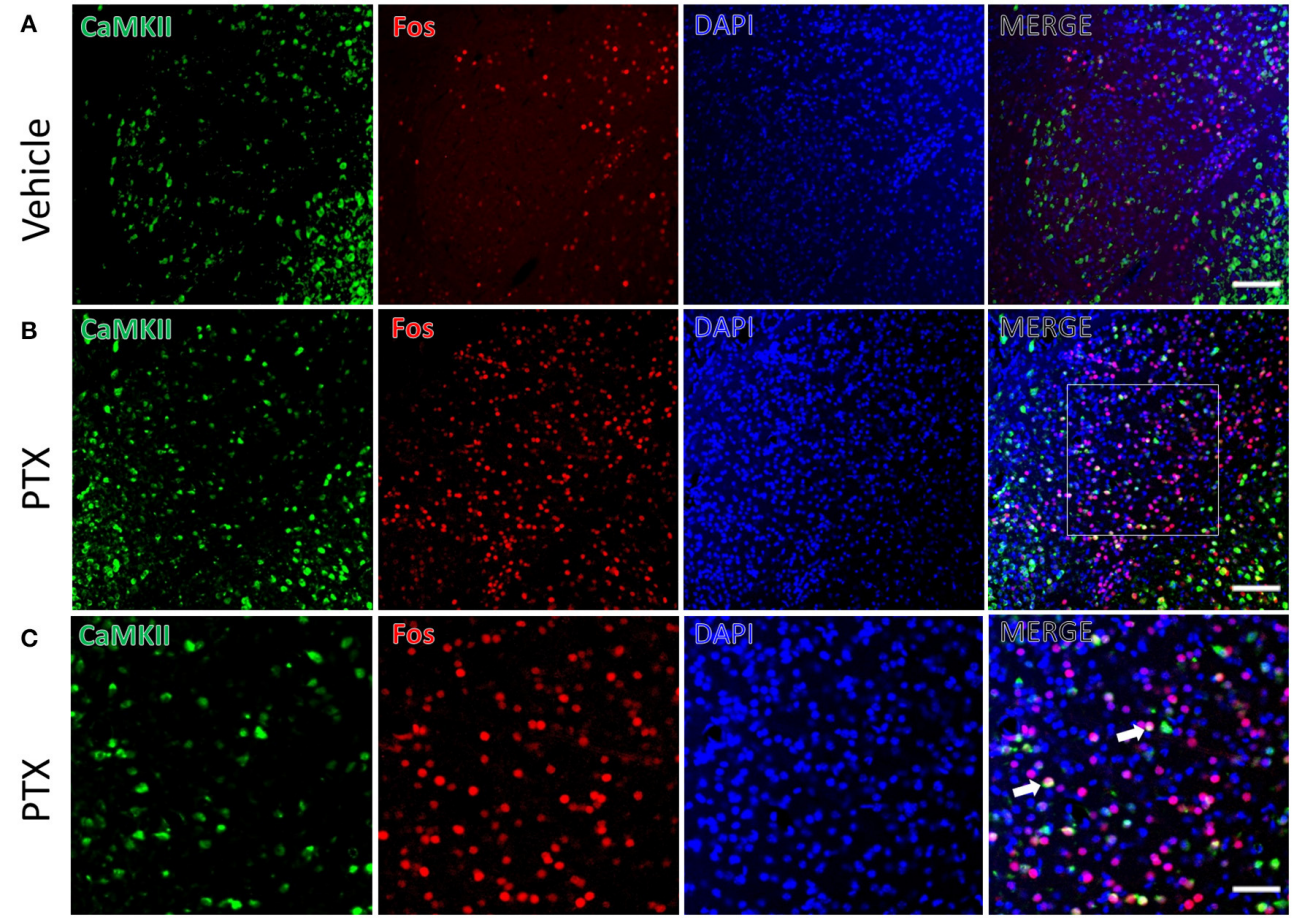
CaMKII neurons in the CeA are activated in paclitaxel-treated mice. **(A)** The expression of CaMKII (green) and Fos (red) in CeA area of the vehicle group. **(B)** The expression of CaMKII (green) and Fos (red) in CeA area in paclitaxel group. **(C)** The magnified images of the rectangles indicated in **(B)**. Arrows indicate part of CaMKII and Fos double-labeled neurons. *n* = 3 mice per group, 2 sections per mouse. Scale bars = 100 μm in **(A)** [applies to **(B)**]; 50 μm in **(C)**.

We used the immediate early gene c-fos to detect changes in neuronal activity in the amygdala in response to paclitaxel treatment. Immunofluorescence staining was performed using mice treated by paclitaxel or vehicle for 9 days. The number of c-fos-positive neurons in the basolateral amygdala of paclitaxel chemotherapy pain model mice (341.00 ± 13.70) was significantly higher than vehicle-treated mice (99.83 ± 8.30, *P* < 0.001, Figures 2A,B). In addition, the number of c-fos-positive neurons in the central amygdala area (62.67 ± 7.02) were significantly higher than that in vehicle-treated mice (33.83 ± 4.66, *P* < 0.001, Figures 3A,B).

### CaMKII Neurons in the Amygdala Are Activated in Paclitaxel-Treated Mice

CaMKII is a marker for excitatory neurons. Paclitaxel-treated mice had more CaMKII and c-fos double-labeled neurons (281.83 ± 8.18) in the BLA compared with that (79.83 ± 6.99) in vehicle-treated mice. The double-labeled neurons in the BLA of the paclitaxel-treated mice accounted for 83.34 ± 4.03% of the c-fos-positive neurons and 68.01 ± 1.50% of the CaMKII-positive neurons (Figure 2). These data suggest that paclitaxel treatment activates CaMKII neurons in the BLA.

The CeA receives projections of CaMKII-positive neurons from the BLA. We also found that there were a large number of CaMKII and c-fos double-labeled neurons in the CeA of paclitaxel-treated mice compared with the control group. CaMKII/FOS double-labeled neuron count (10.50 ± 0.76) in the CeA of the paclitaxel-treated mice was significantly more than that of the control group (3.83 ± 0.74, *P* < 0.001, Figure 3). The double-labeled neurons in the CeA accounted for 17.90 ± 2.51 % of the fos-positive neurons, and 6.67 ± 0.53% of CaMKII-positive neurons in paclitaxel-treated mice. These data suggest that paclitaxel treatment activates CaMKII neurons in the CeA.

### Selectively Inhibiting CaMKII Neurons in the BLA Relieves Paclitaxel-Induced Pain

**Figure 4:**
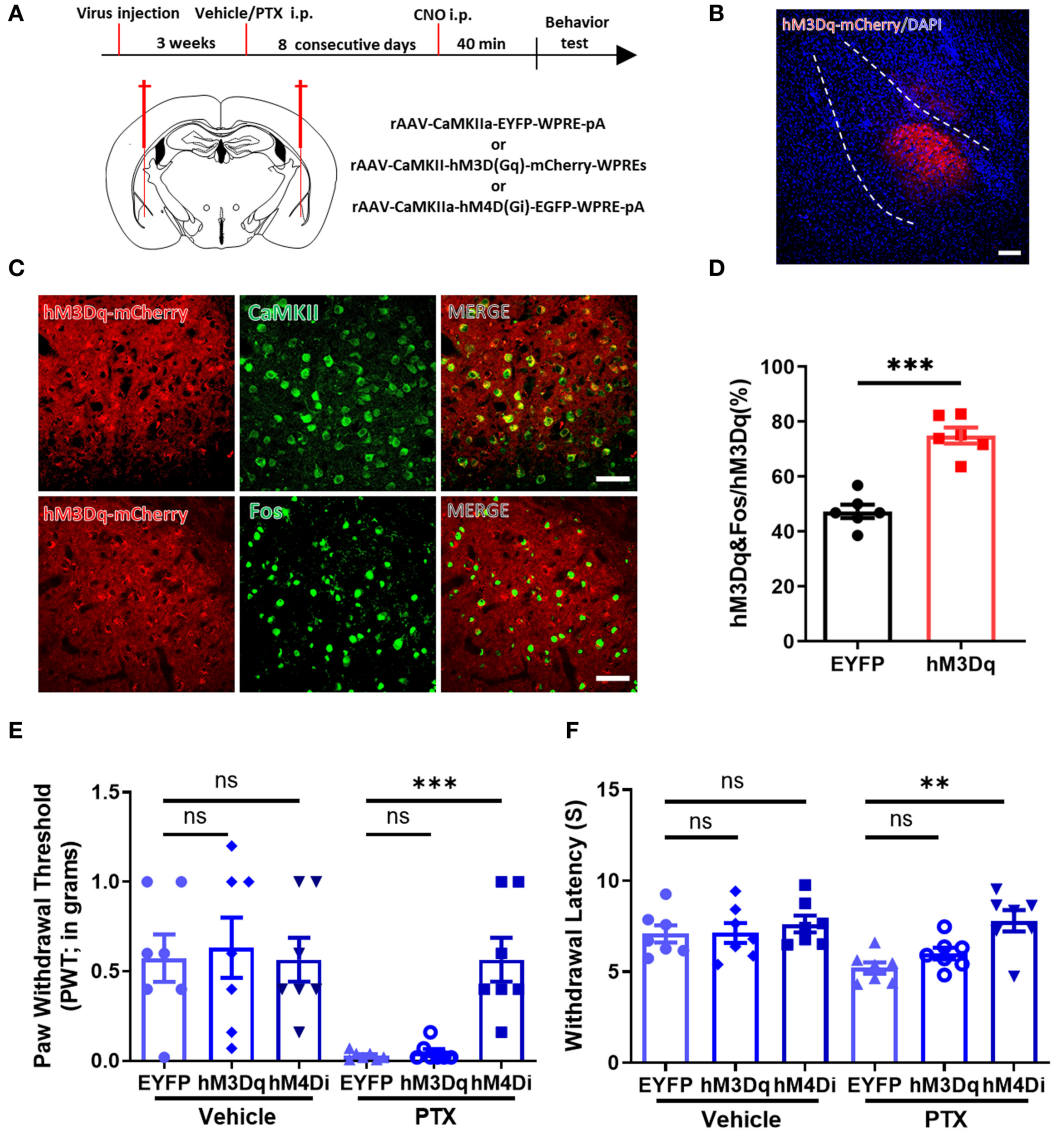
Selectively inhibiting CaMKII neurons in the BLA relieves paclitaxel-induced pain. **(A)** Timeline of virus injection, Clozapine-N-oxide (CNO) administration, and experiments and the Schematic diagram of virus injection site. **(B)** Representative imaging of hM3Dq virus expression within CaMKII neurons in the BLA 3 weeks after viral injection. Scale bars = 100 μm. **(C)** Representative imaging of hM3Dq virus expression within CaMKII neurons in the BLA 3 weeks after viral injection. Under high magnification co-labeled of hM3Dq-mCherry (red) and CaMKII (green), and co-labeled of hM3Dq-mCherry (red) and Fos (green). Scale bars = 50 μm. **(D)** The percentage of the numbers of hM3Dq and Fos co-labeled neurons to hM3Dq labeled neurons in EYFP and hM3Dq. **(E)** Changes in PWT after activation or inhibition of CaMKII neurons. **(F)** Changes in PWL after activation or inhibition of CaMKII neurons. *n* = 6–7, ns, no significant, **P < 0.01, ***P < 0.001.

Because a large number of CaMKII neurons were activated in the BLA in paclitaxel-treated mice, we speculated that manipulating CaMKII neuron activity in the BLA might alter pain behavior. Three vectors rAAV-CaMKIIa-hM4D(Gi)-EGFP-WPRE-pA, rAAV-CaMKIIa-hM3D(Gq)-mCherry-WPREs, and rAAV-CaMKIIa-EYFP-WPRE-pA were injected into the BLA by stereotactic intracerebral injection (Figure 4A). Immunofluorescence staining showed that hM3D-mCherry positive neurons were widely expressed in the BLA (Figures 4B–D). Next, we used the Von Frey test and the Hargreaves test to evaluate the mechanical pain and thermal pain behavior in each group after administration of CNO. Compared with mice expressing EYFP, mice expressing hM4D have significantly higher paw withdrawal thresholds and paw withdrawal latencies. On the other hand, the pain threshold of hM3D-expressing mice did not change significantly (Figures 4E,F). These data suggest that inhibiting CaMKII neurons in the BLA through chemogenetics suppresses mechanical allodynia and thermal hyperalgesia in paclitaxel-treated mice.

### Chemogenetic Inhibition of CaMKII Neurons in BLA Relieves Anxiety-Like Behaviors

**Figure 5:**
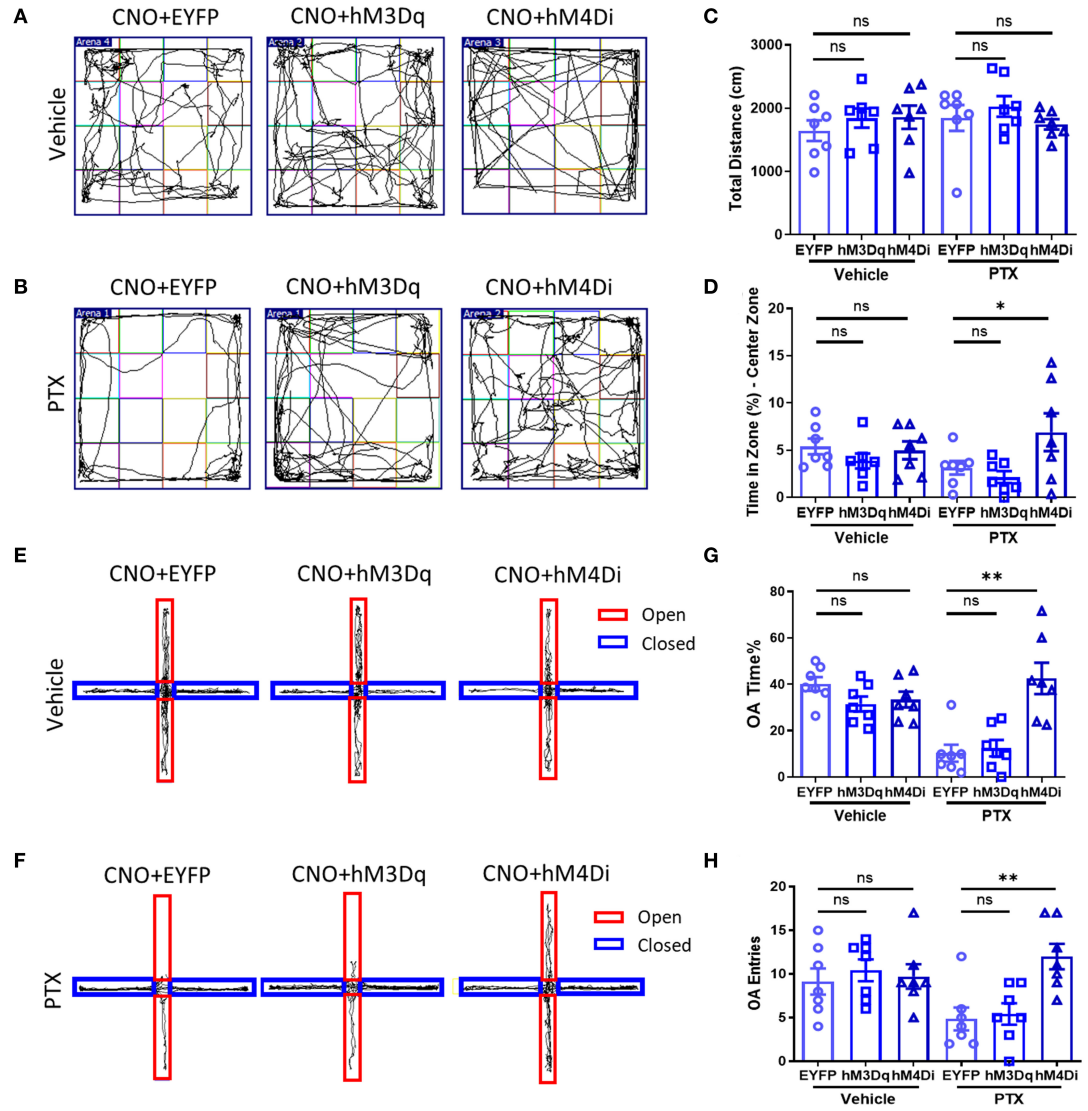
Chemogenetic inhibition of CaMKII neurons in BLA relieves anxiety-like behaviors. **(A,B)** Open field experiment trajectory diagram in vehicle and PTX group after activation or inhibition of CaMKII neurons. **(C)** The total distance in different groups (EYFP, hM3Dq, hM4Di). **(D)** The percentage of central area activity time in different groups (EYFP, hM3Dq, hM4Di). **(E,F)** Elevated plus maze experiment trajectory diagram in vehicle and PTX group after activation or inhibition of CaMKII neurons. **(G)** The percentage of the open arm entry time in different groups (EYFP, hM3Dq, hM4Di). **(H)** The Number of entries open arms in different groups (EYFP, hM3Dq, hM4Di). *n* = 7, ns, no significant, *P < 0.05, **P < 0.01.

As mentioned above, inhibiting CaMKII neurons in the BLA effectively increases the mechanical allodynia and thermal hyperalgesia caused by paclitaxel chemotherapy. Therefore, we speculate that inhibiting CaMKII neurons in the BLA might relieve anxiety emotions in paclitaxel-treated mice. Similarly, using mice injected with rAAV-CaMKIIa-hM4D(Gi)-EGFP-WPRE-pA, rAAV-CaMKIIa-hM3D(Gq)-mCherry-WPREs, rAAV-CaMKIIa-EYFP-WPRE-pA into the BLA (Figure 4A), we performed the open field (Figures 5A,B) and the elevated plus maze experiment (Figures 5E,F) to evaluate anxiety-like behavior after administration of CNO. The total distance in the open field experiment did not significantly differ between these three groups (Figure 5C). However, the percentage of active time in the central area of the hM4D group was significantly higher than that of the hM3D group and the control group (Figure 5D). In addition, in the elevated plus maze experiment, the number of open-arm entry and the percentage of open-arm entry time in the hM4D group were also significantly higher than those in the control group (Figures 5G,H). Compared with the control group, there were no significant differences in the percentage of active time in the central area of the hM3D group (Figure 5D), the number of open-arm entry times, and the percentage of open-arm entry time compared with the control group (Figures 5G,H). Thus, inhibiting CaMKII neurons in the BLA reduced pain and anxiety in paclitaxel treated mice.

## Discussion

Paclitaxel is a chemotherapy drug that widely used to treat many malignant tumors. However, it also causes peripheral nerve damage, neuropathic pain, and negative emotions (2, 9, 42, 43). We found that the CaMKII-positive glutamatergic neurons in the amygdala are involved in the regulation of pain and emotions. By using chemo-genetic approach, selectively inhibiting CaMKII neurons in the BLA produced analgesic effects and effectively relieved the accompanying anxiety in paclitaxel-treated mice.

Previous studies have confirmed that paclitaxel causes a damage of peripheral nerves, including dorsal root ganglia to induce chronic pain (44, 45). As a macromolecular substance, paclitaxel is extremely difficult to pass the blood-brain barrier. Thus, it is most likely that the negative emotions in response to paclitaxel treatment are caused by pain induced by peripheral nerve injury. Anxiety-like emotions generally appear in the early stages of chronic pain (46, 47). In our model, the pain threshold was lowest on the 9th day of paclitaxel injection. At the same time that the pain threshold is significantly reduced, anxiety-like emotions also arise. The chronic pain lasted for more than 21 days.

Recently, “chemobrain” has been proposed because chemotherapy drugs induce negative emotions is called “emotional chemotherapy brain”(48). Previous studies (14) have reported that paclitaxel chemotherapy pain and its negative emotions affect the frontal cortex. CaMKII neurons in the frontal cortex are significantly down-regulated by harmful external stimuli that cause the excitability being transmitted to the amygdala (49). Arthritis pain increases synaptic transmission in the BLA and increases its excitability (50, 51). In our experiment, under the dual effects of chemotherapy pain and negative emotions, compared with the control group, CaMKII neurons in BLA are activated, thus providing evidence that CaMKII neurons in BLA are involved in the occurrence and regulation of pain and related emotions.

The CeA receives the projection of the CaMKII neuron in the BLA (23, 52–54). The excitability of CeA neurons is increased, c-fos and extracellular signal-regulated kinase are activated in visceral pain (55, 56). The increased excitability of CeA in neuropathic pain is accompanied by an increase in expression of corticotrophin-releasing factor and glucocorticoid receptors (57, 58). In our experiment, we observed that some CaMKII-positive neurons were activated in CeA.

In paclitaxel-treated mice, the BLA has more activated CaMKII neurons than other areas of the amygdala. Thus, we determined that the neuropathic pain and negative emotions induced by paclitaxel treatment were regulated by the CaMKII neurons in the BLA by using chemo genetics approach. Our finding is consistent with previous reports (23, 24) showing that a reduction of amygdala activity inhibits pain behaviors. Also, non-NMDA and NMDA receptor antagonists, mGluR1 and mGluR5 antagonists, group II mGluR2/3 agonists and group III mGluR, including mGluR8, CGRP1 and CRF1 receptor antagonists, neuropeptide S activates NPS receptors, and ERK and PKA inhibitors have obvious analgesic effects. It has been reported that the CaMKII neurons in the BLA are activated by conditional fear (27, 59). Our findings in this study suggest that inhibition of CaMKII neurons in the BLA produces significant analgesia and relieves anxiety.

## Conclusion

Therefore, our study showed that the CaMKII neurons in the amygdala participate in the regulation of paclitaxel-induced pain and negative emotions. Inhibition of the CaMKII neurons in the BLA effectively alleviated the pain and negative emotions in paclitaxel-treated mice. These findings provide a new perspective to develop novel approaches for treating pain and negative emotions induced by chemotherapy.

## Data Availability Statement

The raw data supporting the conclusions of this article will be made available by the authors, without undue reservation.

## Ethics Statement

The animal study was reviewed and approved by Fourth Military Medical University.

## Author Contributions

SW, JH, and XW designed the study. JL, DL, JC, JH, GC, YG, GW, and SZ performed the experiments and the data analysis. JL, XW, and SW wrote the manuscript. All authors contributed to the article and approved the submitted version.

## Funding

This study was financially supported by the National Natural Science Foundation of China (82071235 and 81971001) and Natural Science Foundation of Hebei Province (H2021206109).

## Conflict of Interest

The authors declare that the research was conducted in the absence of any commercial or financial relationships that could be construed as a potential conflict of interest.

## Publisher's Note

All claims expressed in this article are solely those of the authors and do not necessarily represent those of their affiliated organizations, or those of the publisher, the editors and the reviewers. Any product that may be evaluated in this article, or claim that may be made by its manufacturer, is not guaranteed or endorsed by the publisher.
