# Supplementary material for: Glutamatergic Neurons in the Amygdala Are Involved in Paclitaxel-Induced Pain and Anxiety
Source: Front Psychiatry. 2022 Apr 14;13:869544. doi: 10.3389/fpsyt.2022.869544 (PMC9049739; doi:10.3389/fpsyt.2022.869544)
Supplement: Supplementary file 1 [file Data_Sheet_1.PDF]

Supplemental Figure 1

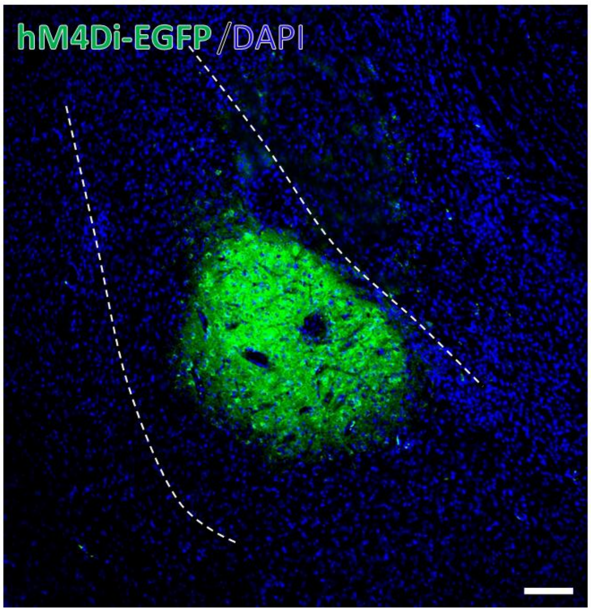

The injection site of hM4Di-EGFP

The representative imaging of hM4Di virus expression in the BLA. Scale bar = 100 µm.
